# Comparative leucocyte populations between two sympatric carnivores (*Nasua narica* and *Procyon lotor*)

**DOI:** 10.1093/conphys/coz050

**Published:** 2019-10-11

**Authors:** Emilio Rendón-Franco, Osvaldo López-Díaz, Oscar Rodríguez-Espinosa, Nora Rojas-Serranía, Roberto Rodríguez-Cabo-Mercado, Maria M B Moreno-Altamirano, Claudia I Muñoz-García, Claudia Villanueva-García, Alvaro Aguilar-Setién

**Affiliations:** 1 Departamento de Inmunología, Escuela Nacional de Ciencias Biológicas, Instituto Politécnico Nacional, Prolongación de Carpio y Calle Plan de Ayala, Miguel Hidalgo, Ciudad de México 11350, México; 2 Departamento de Producción Agrícola y Animal, UAM-Unidad Xochimilco, Calzada del Hueso, Coyoacán, Ciudad de México, 04960, México; 3 Facultad de Medicina Veterinaria y Zootecnia, Universidad Nacional Autónoma de México, Ciudad Universitaria, Avenida Universidad, Coyoacán, Ciudad de México 04510, México; 4 División Académica de Ciencias Biológicas, Laboratorio de Ecología del Paisaje y Cambio Global, Universidad Juárez Autónoma de Tabasco, Carretera Villahermosa-Cárdenas, Centro, Villahermosa, 86150, México; 5 Unidad de Investigación Médica en Inmunología, Centro Médico Nacional Siglo XXI, Instituto Mexicano del Seguro Social, Avenida Cuauhtémoc, Cuauhtémoc, Ciudad de México, 06720, México

**Keywords:** carnivore, leucocyte, *Nasua narica*, phagocytosis, *Procyon lotor*

## Abstract

Coatis (*Nasua narica*) and raccoons (*Procyon lotor*) potentially play an important role in zoonotic diseases because they may carry pathogens and can transmit them to humans. To date, our understanding of the immune function of these two carnivores is deficient. The aim of this study was to compare the number of leucocyte subtypes and the phagocytic capacity between the coati and the raccoon. Blood samples were collected, and leucocyte subtypes were characterized and counted by flow cytometry and microscopy, respectively. Phagocytosis was analysed by kinetic assay. Differences in leucocytes between these two species were found; the total count of neutrophils was higher in raccoons than in coatis, but lymphocytes and eosinophils were higher in coatis than in raccoons. Antigen reduction was more rapid for the coatis. However, raccoons had a higher efficient endocytic process than coatis. This study provides the basis for understanding the procyonid immune system, which informs conservation, particularly since some procyonids are imperilled.

## Introduction

Coatis (*Nasua narica*) and raccoons (*Procyon lotor*) are carnivores highly adapted to human-altered environments (Lotze and Aderson, 1979; Gompper, 1995). Carnivores play an important role in ecosystems because they suppress populations of prey and their pathogens (Ostfeld and Holt, 2004). In addition, the composition of species in an ecosystem is an important factor for pathogen persistence so it is important to understand inter-specific differences in immune function (Keesing *et al*., 2010; Suzán *et al*., 2015). Procyonids are a family of carnivores that are naturally distributed in America, but they have also been introduced in Europe and Asia (Koepfli *et al*., 2007; Alda *et al*., 2013). Two of the widely distributed procyonids are the raccoon and the coati, which have mostly sympatric ranges across the American continent (Lotze and Aderson, 1979; Gompper, 1995). Raccoons have been implicated as reservoir for several diseases such as rabies, leptospirosis, salmonellosis, tularemia and baylisascariasis (Compton *et al*., 2008; Lembo *et al*., 2008; Recuenco *et al*., 2008; Yeitz *et al*., 2009; Beltrán-Beck *et al*., 2012; Duncan *et al*., 2012). There are few reports of diseases such as rabies, Chagas disease, salmonellosis and *Orthopoxvirus* in coatis (Aréchiga-Ceballos *et al*., 2010; Martínez-Hernández *et al*., 2014; De-la-Rosa-Arana *et al*., 2016; Gallardo-Romero *et al*., 2016; Martínez-Hernández *et al*., 2016); however, their role as a reservoir is unclear, partly because their immune response has been poorly studied. Understanding the pathogen–immune system relationship is important in areas where these carnivores are sympatric with other imperilled carnivores such as jaguar (*Panthera onca*), pygmy raccoon (*Procyon pygmaeus*) and insular raccoon (*Procyon insularis*). Indeed, all of them are facing a high risk of extinction, among other reasons because of the occurrence of outbreaks of infectious diseases, such as distemper virus (Mena, 2007; Nava *et al*., 2008).

Leucocyte profiles (leucocyte subpopulations) have been used as a reflex of stress and as an outcome disease predictor in different groups of vertebrates (Davis *et al*., 2008). However, leucocytes’ baseline and its comparison among species are still poorly evaluated. In this context, it is important to characterize and understand differences in the immune structure and response among species, particularly in species that are sympatric and have close phylogenetically relationships.

Studies in coatis and raccoons have shown different humoral and cellular immune responses (Gallardo-Romero *et al*., 2016; Martínez-Hernández *et al*., 2016). However, the study of immune structure and function, even in the most basic aspects, has been poorly evaluated and compared. The objective of this study was to compare the leucocyte subpopulations and their function between two sympatric carnivores of the species *N. narica* and *P. lotor*, in order to use this information as reference for understanding of basic immunology in this and other imperilled procyonids.

## Materials and methods

### Animal population, capture and sampling

Both species, *N. narica* and *P. lotor*, inhabit in the Arqueo-Ecological Park ‘Parque Museo de la Venta’ in Villahermosa, Tabasco, in Southeastern Mexico. The park is located in the centre of the city and is surrounded by urban infrastructure and a lagoon (see Supplementary Figure 1). The park has 3 ha of vegetation (secondary forest) and a zoological area. Both coatis and raccoons roam free in the park and surrounding areas, choose freely their food from forest resources and sometimes are supplemented by park staff with some variety of fruits, boiled eggs, whole-meal bread and oatmeal. The estimated numbers of adults in both populations were approximately 108 coatis and 98 raccoons, with some fluctuations across time (Martínez-Hernández *et al*., 2014).

Animals were trapped in January 2018 by two different methods. Coatis were captured using a tranquilizer dart fired with a blowpipe which was used to sedate the animals; for raccoons, Tomahawk® traps were placed at noon and baited with sardines embedded in tomato sauce. All traps were checked the following morning; raccoons captured were chemical-restrained. Chemical restraints of both species were done using a combination of ketamine–xylazine following the previously described procedure of Martínez-Hernández *et al.* (2014). Once restrained, vital signs of animals were monitored. Next, 5 mL of blood was taken directly from the external jugular vein and preserved in citrate (cytometry and phagocytosis assay) and EDTA (Leucogram). The restraint and handling of each animal lasted for about 30 min. Samples were kept at 4°C until they were analysed in the lab (about 48 to 72 h after). Animals were released in the park within 6 h after recovery from anaesthesia. All sampling was done under the permission of the Mexican Ministry of Environment and Natural Resources (FAUT-0250) and The Ethics committee of the Universidad Autonoma Metropolitana (DCBS.CICUAL.00813).

### Comparison of leucocytes by size and complexity between raccoons and coatis

Blood samples of 10 individuals, five of each species, were used to analyse the size and complexity of leucocytes by flow cytometry assay. To do so, 100 μL of blood was put into 1.5-mL Eppendorf® microtubes. Erythrocytes were lysed with 500 μL distilled water and mixed gently, then 500 μL of KCl was added and mixed again. Cells were centrifuged at 1500 rpm for 10 min, and the supernatant was decanted and the pellet was resuspended in saline phosphate buffer (PBS) and centrifuged again to wash the cells twice. Cells were fixed in paraformaldehyde 4% for 30 min, washed and resuspended in 400 μL PBS.

Cells were evaluated by flow cytometry using a FACS Calibur Flow cytometer using CellQuest® software. Ten thousand events were recorded, and areas compatible by size and complexity with granulocytes, monocytes and lymphocyte were gated. Percentages of events in the gate were evaluated for each individual.

### Comparison of leucocyte differential count between raccoons and coatis

Leucocyte differential count comparison was performed on all captured animals, 29 coatis and 9 raccoons, by conventional techniques (Thrall *et al.*, 2012). From total blood with EDTA, erythrocytes were lysed by Turk’s solution Hycel®. The white blood cell count was made in a manual hemocytometer. A differential leucocyte count was done by Romanowsky staining with oil magnification (×100). The total count of each cell line was calculated by the following formula (percentage of cell line * white blood cell count/100) = total cell line in ×10^9^/L.

### Comparison of phagocytosis between raccoons and coatis

Twelve individuals, six of each species, were evaluated for the phagocytic capacity at different times; this evaluation was repeated five times at 1, 15, 30, 45 and 60 min. The procedure was as follows: 400 μL of complete blood sample with citrate was placed over the glass slide, previously cleaned and sterilized and placed in a 12-well culture dish. Samples were incubated for 1 h at 37°C, 5% CO_2_ and 95% humidity, in order to attach the phagocytic cells to the glass. After the incubation period, two washes were done with PBS to eliminate erythrocytes and non-adherent leucocytes. One millilitre of Dulbecco’s Modified Eagle’s medium (DMEM) without foetal bovine serum, 400 μL of 10^7^ yeasts/mL in PBS and 250 μL of tetrazolium blue (NBT) 0.5% in saline physiologic solution (SSF) were added to each sample. After the allocated time, samples were washed twice with PBS and stained with 0.5% safranin for 30 min; after that, the glass slide was washed once with tap water. The slides were mounted in Entellan (Merk®) medium and were examined under the immersion objective. Values were obtained by averaging the results of the analysed samples in which up to 100 cells per slide were counted. When cells had only one yeast inside, they were categorized in three stages: cells in endocytosis (with one yeast inside), cells in reduction (with one yeast in reduction noted by a blue colour) and cells in degradation (with fragments of yeast in reduction). The stage was only recorded for cells with one yeast phagocyted, because cells with more than one yeast had different stages at the same time. Additionally, for each cell the number of yeast particles inside each cell was recorded. All observations were carried out by the same person (E.R.F.).

## Data analysis

Differences in means of each cell line, proportions of cells in phagocytosis and the percentage of cell lines in flow cytometry according to procyonid species were analysed with the Mann–Whitney test using Past® 3.14 software (Hammer *et al.*, 2001). Significance was considered for *P* < 0.05.

## Results

### Comparison of leucocytes by size and complexity between raccoons and coatis

**Figure 1:**
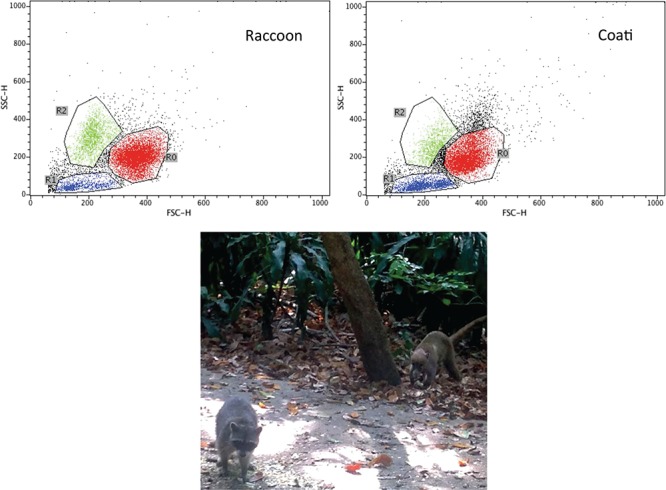
Representative dot plot of flow cytometry of size versus complexity. Red dots (R0) represent monocytes, green dots (R2) granulocytes and blue dots (R1) lymphocytes. The lines around dots denote the gates of the leucocyte subpopulation

Slight differences were noticed between species, where coatis had a higher percentage of lymphocytes than raccoons (18.7 ± 4.2 vs 11.4 ± 1.2), but raccoons had a higher percentage of granulocytes (54.6 ± 5.7 vs 48.9 ± 2.2) and monocytes (17.8 ± 4.4 vs 14.1 ± 2.6). However, none of the differences were statistically significant (*P* > 0.05). Figure 1 shows a representative scatter plot of each species. Particularly, one coati showed a shift in lymphocyte and monocyte proportion suggesting an infection process. This was associated with leucopoenia due to strong lymphopenia and mild monocytopenia, suggesting a viral infection. After removing this individual from the analysis, the difference in lymphocyte percentage between raccoons and coatis was statistically significant (21.6 ± 3.8 vs 11.4 ± 1.2; *P* = 0.01).

### Comparison of leucocyte differential count between raccoons and coatis

All leucocyte types were detected, although band neutrophils in both species and basophils in raccoons were absent. Total leucocyte count did not differ between species (*P* = 0.127), despite that raccoons had a slightly higher total count (Table 1). However, when a specific leucocyte type was compared, some differences were detected. Neutrophils were higher in raccoons than in coatis (*P* < 0.001). Lymphocytes and eosinophils were higher in coatis than in raccoons (*P* = 0.0004 and *P* < 0.001, respectively). Slight differences were detected in monocytes and basophils, but were not statistically significant (*P* = 0.513 and *P* = 0.807, respectively).

**Table 1:** Leucocyte differential counts

	Coatis *n* = 27 Mean (±SE)	Raccoons *n* = 9 Mean (±SE)	*P* value
Leucocytes	9.45 (±0.44)	12.14 (±1.78)	*P*=0.1274
Neutrophils	4.39 (±0.39)	10.19 (±1.50)	*P*=0.0001
Lymphocytes	3.71 (±0.28)	1.55 (±0.34)	*P*=0.0004
Monocytes	0.40 (±0.06)	0.30 (±0.07)	*P*=0.5127
Eosinophils	0.93 (±0.11)	0.09 (±0.06)	*P*=0.0001
Basophils	0.01 (±0.01)	0 (na)	*P*=0.8066

All counts were expressed in cell ×10^9^/L. SE = standard error; na = not applicable

Since sex is known to influence immunological responses, comparisons between sexes of the same species were done. The only difference between males and females found were the neutrophils from coatis (Supplementary Figure 1). However, when neutrophils were compared between species stratified by sex, differences between species were still apparent (data not shown).

### Comparison of phagocytosis between raccoons and coatis

**Figure 2:**
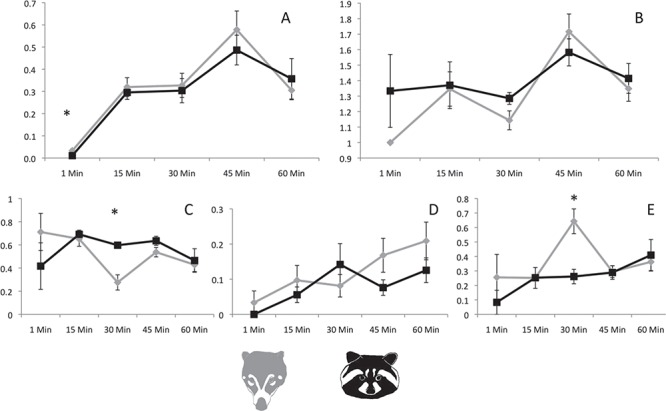
A. Proportions of cells in phagocytosis. B. Average of phagocyted yeast. C. Proportions of cells with ingested yeast. D. Proportions of cells with reduced yeast. E. Proportions of cells with degraded yeast. Asterisks show significant differences between species (*P* < 0.05), and error bars represent the standard error.

For both species, the percentage of cells in phagocytosis increased from minute 1 to the highest point at minute 45, then fell at minute 60 (Fig. 2A). Coatis had slightly higher percentages from minute 1 to minute 45 than raccoons, but differences were only statistically significant at minute 1 (*P* = 0.039).

When different stages of phagocytosis were compared between species, raccoons showed a more efficient endocytosis process than did coatis at minute 30 (*P* = 0.004), but coatis showed a more efficient degradation process than did raccoons at the same time (*P* = 0.004, Fig. 2C–E).

Finally, for coatis the number of yeasts per phagocytic cell showed a range from 1 to 13 yeasts per cell and an average from 1 to 1.71 yeasts per cell for the four evaluation times (1, 15, 30 and 60 min) of the experiment. For raccoons, the range was from 1 to 12 yeasts per cell and an average from 1.30 to 1.63 yeasts per cell. No significant differences were detected (*P* > 0.05, Fig. 2B).

## Discussion

Cellular subpopulations described for size and complexity of this study are similar to other domestic carnivores previously reported by Joetzke *et al.* (2012). For example, monocyte and lymphocyte differences have been reported between domestic dogs and raccoons (Heinrich *et al.*, 2015). However, these animals belong to different families, Canidae and Procyonidae respectively, so this is the first time that differences in the cellular subpopulation composition by size and complexity were detected between two sympatric and closely related species of the Procyonidae family. The findings of our study could explain some of the differences found by other authors in coatis’ and raccoons’ immune response to pathogens (Martínez-Hernández *et al*., 2014; Gallardo-Romero *et al.*, 2016; Martínez-Hernández *et al.*, 2016). However, the detection of specific cell lineages (i.e. CD3, CD4, CD8, CD14, CD79a, MHC II) can only be achieved through the standardization of its lineage-specific markers. Molecular markers of those lineages exist but are rodent- and human-specific, so they will not work for carnivores (Heinrich *et al.*, 2015).

In carnivores, complete blood count has been used as an individual screening tool for detection of subclinical effects of diseases; however, interspecific comparisons are lacking. Some studies in carnivores showed that body mass, sexual maturity, gestation, group size and mating partners correlate (positively or negatively) with total leucocyte and neutrophil counts. Other leucocyte correlations identified were lymphocytes associated with population density and eosinophils with proportion of meat in the diet (Nunn *et al.*, 2003). In our study, we also found differences in neutrophils (higher value in raccoons), and lymphocytes and eosinophils (higher value in coatis), but it should be noted that both species share similarities such as body mass, sexual maturity, gestation and mating partners. Consequently, differences in lymphocytes and eosinophils detected between these procyonid species may be explained by other characteristics associated with the species such as group size, density and percentage of meat in diet, which according to literature are all higher in coatis (Lotze and Aderson, 1979; Gompper, 1995). Nevertheless, the findings related to neutrophils require a more detailed study given the ecological variables to consider, for example aggressiveness and interspecific interaction among others (Nunn *et al.*, 2003).

Despite the fact that phagocytosis has been evaluated in carnivores, no comparison has been made, probably due to the difficulty of capturing carnivores in similar environmental circumstances. For dolphins (*Tursiops truncatus*), the highest reduction of NBT was after 30 min of incubation, which corresponds to the highest degradation time of coatis but not raccoons. The same study found that the optimal time for evaluation of phagocytosis was 12 h; however, they used latex particles that are non-degradable (Noda *et al.*, 2003). This contrasts with our assessment where the 1-h assay, under the previously mentioned conditions, was sufficient for phagocytosis assessment.

Differences in phagocytosis time among species were not previously reported for carnivores. Coatis have a faster rate of yeast degradation, suggesting a more efficient phagocyte cells which could explain the lower number of phagocytes. However, more assays should be performed using other antigens as processing ovalbumin or bacteria intake in order to evaluate phagocyte cells.

This study presents an important approach to evaluate differences in the immune system; however, the assay should be also assessed in other contexts including across seasons where natural pathogens are present, since variations in the complete blood cell count have been associated with seasonality (Martínez-Hernández *et al.*, 2016). Also, specific markers for lineage should be assessed for more specific leucocyte subpopulation evaluation; however, to date there are no specific or validated markers for coatis.

Differences in leucocyte subpopulations of coatis can be explained by their high contact rates among individuals, because coatis have greater social cohesion when compared to raccoons. They are also more aggressive between them and have a characteristic mating and breeding system that favours the contact among individuals. The aforementioned interactions could also help to explain the differences in the detection of pathogens identified in other studies of these two species. It is important to further evaluate these and other immunological characteristics and also the pathogens’ carrying capacity between these closely related species, in order to elucidate its reservoir competence explained through their immunological resistance against pathogens.

## Supplementary Material

suppl_data_coz050
