# Supplementary material for: Comparative leucocyte populations between two sympatric carnivores (Nasua narica and Procyon lotor)
Source: Conserv Physiol. 2019 Oct 11;7(1):coz050. doi: 10.1093/conphys/coz050 (PMC6788493; doi:10.1093/conphys/coz050)
Supplement: suppl_data_coz050 [file suppl_data_coz050.pdf]

Supplementary figure. Schematic map of sampling site that shows the whole Mexican county (upper figure with Tabasco state in the southeastern orange-coloured), Tabasco state and in the vertex Parque Museo de la Venta inside of Villahermosa city (lower left figure orange-coloured) and Parque Museo de la Venta (lower right figure). Parque Museo de la Venta (green) is surrounded by urban areas (grey) and a lagoon (blue), and coati and raccoon (black) and Olmec colossal heads (light grey) are seen inside the park.

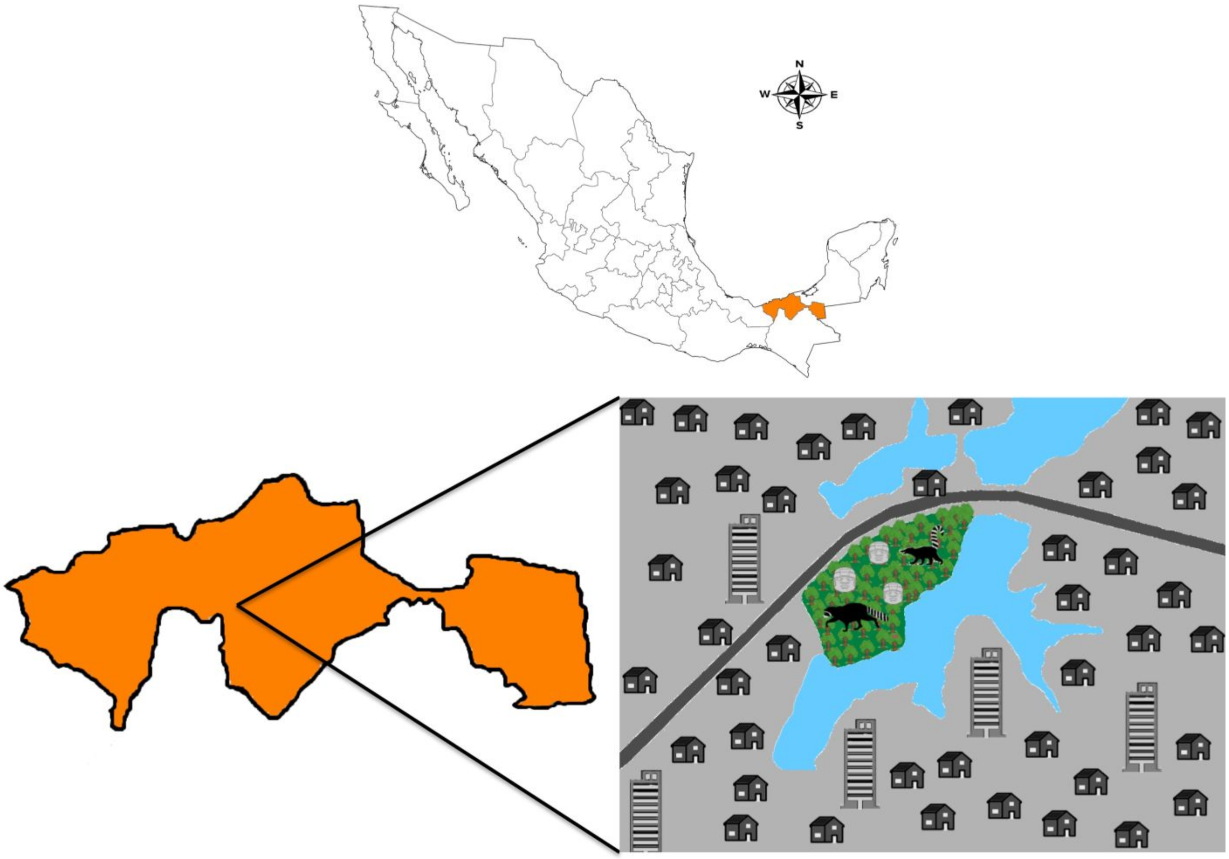

Supplementary table. Species and sex differences in leukocytes differential counts.

	Coatis			Raccoons		
	females n=9	males n=18		females n=5	males n=4	
Leukocytes	9.20 (±0.67)	9.57 (±0.57)	p=0.8169	10.37(±1.29)	14.35(±3.63)	p=0.5402
Neutrophils	3.30 (±0.38)	4.93(±0.51)	p=0.0476	8.46(±1.37)	12.34(±2.74)	p=0.1779
Lymphocytes	4.37(±0.44)	3.37(±0.34)	p=0.0537	1.46(±0.07)	1.65(±0.83)	p=0.2683
Monocytes	0.57(±0.11)	0.31(±0.05)	p=0.0536	0.26(±0.06)	0.35(±0.14)	p=0.9025
Eosinophils	0.91(±0.11)	0.93(±0.15)	p=0.6250	0.15(±0.10)	0(na)	p=0.1094
Basophils	0.02(±0.02)	0(na)	p=0.6103	0(na)	0(na)	na

All counts were expressed in cell x10^9/L, na= not applicable
